# Low-Dimensional Motor Cortex Dynamics Preserve Kinematics Information During Unconstrained Locomotion in Nonhuman Primates

**DOI:** 10.3389/fnins.2019.01046

**Published:** 2019-10-04

**Authors:** David Xing, Mehdi Aghagolzadeh, Wilson Truccolo, Erwan Bezard, Gregoire Courtine, David Borton

**Affiliations:** ^1^School of Engineering, Brown University, Providence, RI, United States; ^2^Department of Neuroscience, Brown University, Providence, RI, United States; ^3^Carney Institute for Brain Science, Brown University, Providence, RI, United States; ^4^U.S. Department of Veterans Affairs, Center for Neurorestoration and Neurotechnology, Providence, RI, United States; ^5^CNRS, Institut des Maladies Neurodégénératives, UMR 5293, Bordeaux, France; ^6^Université de Bordeaux, Institut des Maladies Neurodégénératives, UMR 5293, Bordeaux, France; ^7^Center for Neuroprosthetics and Brain Mind Institute, School of Life Sciences, Swiss Federal Institute of Technology (EPFL), Geneva, Switzerland; ^8^Department of Clinical Neuroscience, Lausanne University Hospital (CHUV), University of Lausanne (UNIL), Lausanne, Switzerland; ^9^Defitech Center for Interventional Neurotherapies (NeuroRestore), CHUV/UNIL/EPFL, Lausanne, Switzerland; ^10^Department of Neurosurgery, CHUV, Lausanne, Switzerland

**Keywords:** low dimensional dynamics, locomotion, non-human primate (NHP), poisson linear dynamical system, primary motor cortex (M1)

## Abstract

The dynamical systems view of movement generation in motor cortical areas has emerged as an effective way to explain the firing properties of populations of neurons recorded from these regions. Recently, many studies have focused on finding low-dimensional representations of these dynamical systems during voluntary reaching and grasping behaviors carried out by the forelimbs. One such model, the Poisson linear-dynamical-system (PLDS) model, has been shown to extract dynamics which can be used to decode reaching kinematics. However, few have investigated these dynamics, especially in non-human primates, during behaviors such as locomotion, which may involve motor cortex to a lesser degree. Here, we focused on unconstrained quadrupedal locomotion, and investigated whether unsupervised latent state-space models can extract low-dimensional dynamics while preserving information about hind-limb kinematics. Spiking activity from the leg area of primary motor cortex of rhesus macaques was recorded simultaneously with hind-limb joint positions during ambulation across a corridor, ladder, and on a treadmill at various speeds. We found that PLDS models can extract stereotyped low-dimensional neural trajectories from these neurons phase-locked to the gait cycle, and that distinct trajectories emerge depending on the speed and class of behavior. Additionally, it was possible to decode both the hind-limb kinematics and the gait phase from these inferred trajectories just as well or better than from the full neural population (18-80 neurons) with only 12 dimensions. Our results demonstrate that kinematics and gait phase during various locomotion tasks are well represented in low-dimensional latent dynamics inferred from motor cortex population activity.

## 1. Introduction

With the advent of large scale intracortical recordings allowing for the simultaneous interrogation of dozens to hundreds of neurons, the study of the role of motor cortex in the generation of movement has been steadily moving toward investigation of cortical dynamics in the brain on the population level. Ensembles of cortical neurons are organized as recurrently connected networks, introducing shared variability among the constituent cells, in turn constraining firing activity to a lower-dimensional space (Yu et al., 2009; Truccolo et al., 2010; Afshar et al., 2011; Churchland et al., 2012; Cunningham and Yu, 2014; Sadtler et al., 2014; Gallego et al., 2017; Pandarinath et al., 2018). Dimensionality reduction techniques can be used to extract the coordinated neural activity of a population, and reveal structures that may be hidden at the isolated single-neuron level. Previous investigations have shown that for reaching movements with the arm, semi-oscillatory dynamics, inferred using jPCA, are a common feature underlying a variety of different reach movements (Churchland et al., 2012). Additionally, a common low-dimensional neural manifold underlying various wrist movements have be found using demixed principle component analysis, or dPCA (Gallego et al., 2018).

Besides dimensionality reduction, many of these techniques also employ dynamical systems models. These models address how the values at the current time step depend on the values at previous time steps, usually in the form of a temporal transition matrix. Poisson Linear-Dynamical-System (PLDS) is one such technique which employs both unsupervised dimensionality reduction as well as explicit temporal dynamics. PLDS maps low-dimensional latent states to the measured high-dimensional neural spiking activity through an observation point-process model and explicitly estimates the dynamics of these latent states as it evolves through time with a linear mapping (Truccolo et al., 2005; Macke et al., 2011). Unlike PCA and dPCA, PLDS models the low-dimensional space as a state-space in a linear dynamical system, thereby explicitly accounting for the temporal relationships in the population. Although these low-dimensional dynamics are inferred through an unsupervised process, they are able to retain relevant behavioral information. In reaching behaviors, explicit state-space models similar to PLDS have been shown to increase closed-loop BMI performance in cursor-control tasks (Kao et al., 2015) and decoding accuracy during forelimb reaching behaviors (Aghagolzadeh and Truccolo, 2014, 2016), demonstrating that only a small number of dimensions from the neural population space is needed to capture the movement kinematics during voluntary forelimb movements.

However, reaching actions with the arm are typically highly precise and have a strong voluntary control component, whereas locomotion movements have a higher degree of autonomy. For example, in felines, injection of the neurotoxins or ablation of the motor cortex does not affect the ability of the animal to walk along a flat surface, although their ability to step over a ladder and over obstacles is impaired (Beloozerova and Sirota, 1988; Drew et al., 1996). These results suggest that motor cortex may play a less active role in the control of limb movements during basic, unobstructed locomotion compared to movements that require top-down voluntary control. In non-human primates, inducing corticalspinal tract lesions showed that although there is some locomotion deficits post-lesion, these recover quickly, while dexterous foot grasping remain severely impaired, even after 3 months post-lesion (Courtine, 2005).

These findings suggest that the contribution of M1 to the control of movements might be different during locomotion compared to during reaching, although how exactly the role of M1 is changing between these two behaviors is still not well understood. It has been well known for several decades that M1 is active during walking, and that cortical neurons are phasically tuned to the gait cycle (Drew, 1988; Beloozerova and Sirota, 1998; Drew et al., 2008). Yakovenko and Drew recorded from corticospinal neurons during reaching and during walking over an obstacle in cats and found that the firing onset phase of certain neurons correlated with the onset phase of muscle activation for both types of movements (Yakovenko and Drew, 2015), suggesting similar encoding of movements in M1 for both types behaviors. However, a recently published study in mice found that the population-level structure of M1 neurons is disparate during reaching and lever pulling compared to simple treadmill walking (Miri et al., 2017). These contrasting conclusions demonstrate that there is still a lack of consensus on the role of motor cortex during locomotion-related activities, and it is still unclear whether M1 is contributing to the control of the limbs in a similar manner during walking as during directed reaching movements. In particular, it has yet to be shown whether the latent state-space models that capture movement parameters during precise arm reaching would also be able to capture hind-limb movements during the potentially less engaging act of locomotion. While previous studies have found low dimensional representations of motor cortex activity in non-human primates during simple treadmill walking (Foster et al., 2014; Yin et al., 2014), they have not shown that movement kinematics are preserved in these dynamics.

**Figure 1:**
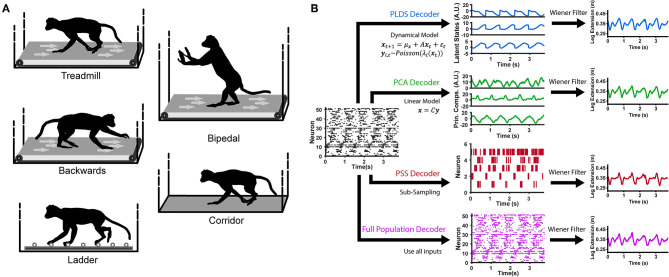
Locomotion behaviors and neural decoding with latent state models. **(A)** Animals were trained to perform different locomotion tasks in a freely moving, untethered environment. The tasks include basic treadmill walking at various speeds, backwards treadmill walking, bipedal treadmill walking, corridor walking, and ladder walking. **(B)** Construction of the neural decoders. For decoders implementing dimensionality reduction (PLDS, PCA, PSS), rasters of neural spike counts for each gait cycle (left plot) undergo either an orthogonal linear transformation to extract principle components (center, top), a count-process filter based on a state-space model to extract low-dimensional latent trajectories (center, middle) or a greedy search algorithm to obtain an optimal subsample of neurons (center, bottom). These low-dimensional features are then used as inputs to a Wiener filter for reconstructing the estimate of a desired locomotion variable, such as the leg extension distance (right). The Full Population decoder (bottom path) performs decoding directly on all recorded normalized neural spike counts, binned at 100 ms. For decoding kinematics and gait, the trials of all tasks were combined and shuffled for each session.

Here, we aim to determine whether the PLDS latent-state model is able to extract low-dimensional dynamics which are informative of the limb movements. We define informative as having the ability to decode hind-limb joint kinematics as well as gait phase, during various locomotion tasks such as treadmill, corridor, and horizontal ladder walking (Figure 1A). To test this, we used either firing rates of the full recorded population of single neurons or the corresponding low dimensional dynamics as input features into a Wiener filter decoder which attempts to reconstruct the kinematics from these inputs. We demonstrate that for PLDS inputs, only a small number of dimensions are necessary to decode limb kinematics and gait phase as accurately as the full neural population. As far as we are aware, this is the first employment of explicit state-space models during both basic locomotion along a treadmill and corridor, as well as during directed locomotion along evenly and unevenly spaced ladders in nonhuman primates. Furthermore, it is also the first demonstration that cortical ensemble dynamics robustly captures behavioral information such as limb kinematics and gait phase during these different ambulatory behaviors.

## 2. Methods

### 2.1. Surgery

Five male rhesus macaques between 5 and 8 years of age were implanted with 96-channel microelectrode arrays (Blackrock Microsystems, UT) in one hemisphere of the leg area of primary motor cortex (M1), located medially along the precentral gyrus (He et al., 1993). The details of the surgical implantation procedures have been described previously (Yin et al., 2014). Experiments complied with the European Union directive of September 22, 2010 (2010/63/EU) on the protection of animals used for scientific purposes in an AAALAC-accredited facility following acceptance of study design by the Institute of Lab Animal Science (Chinese Academy of Science, Beijing, China). Experiments were approved by the Institutional Animal Care and Use Committee of Bordeaux (CE50) under the license number 50120102-A.

### 2.2. Kinematic Data Collection

To obtain kinematic data, white reflective markers were painted over the shaved skin overlaying the right iliac crest (crest), greater trochanter (hip), lateral condyle (knee), lateral malleolus (ankle), 5th metatarsophalangeal (metatarsal), and outside tip of the fifth digit (toe tip). Marker locations were identified by feeling for the bony projections of the above anatomic landmarks under the skin; see Capogrosso et al. (2016) for a visualization of the marker locations. Videos were captured at 100 Hz from four high-speed cameras placed around the enclosures. Motion tracking software (Simi motion systems, Germany) was used to determine the 3D spatial coordinates of each marker, after calibrating the cameras in 3D space using known calibration objects at the beginning and end of each session. The origin of the coordinate system was set to the iliac crest of the animal. The horizontal axis was set to the direction of walking, while the vertical axis was set to the height off the ground. Joint angle was set as the inner angle between the two limb segments. The polar nature of the joint angles can confound error calculations (for example, 0° is closer to 350° than 180°, although 350–0 > 180–0), so instead of decoding the joint angles directly, we decoded the sine and cosine of each angle. Additionally, the total leg extension was calculated as the distance between the iliac crest and the metatarsal joint. Neural data, sampled at 30 kHz, was synchronized with the kinematic data using a camera-start trigger signal at the start of each trial of each recording session.

### 2.3. Tasks

Subjects carried out various behavioral motor tasks (Figure 1A). Monkeys were trained to walk in an enclosed treadmill at speeds of 1.1, 1.6, 2.4, 3.2, 4.0, 4.8, and 6.4 km/h (TRM trials). The enclosure was approximately one and a half meters long by one meter high by half a meter wide. It was constructed from clear Plexiglas, which is both visually and RF transparent. Spontaneously, they would switch to a bipedal gait, or start walking backwards (BIP and BACK trials, respectively). In addition, monkeys walked along a straight 3 m long corridor over a flat surface (CORR trials) and over a horizontal ladder with rungs either evenly spaced 35 cm apart or irregularly spaced (LAD trials). These tasks were self paced, with the average corridor walking speed at 3.24 km/h (0.79 km/h standard deviation) and the average ladder walking speed at 3.49 km/h (0.79 m/s standard deviation). Monkeys were trained for at least 1 month to walk to the end of the corridor or ladder to receive a food reward in response to an auditory beep and flash of light.

Recording was carried out over 1–2 days for each monkey. Each day consisted of recording different randomly interleaved sessions for each task. Each treadmill session consisted of approximately 1 min of walking. These sessions were divided into epochs which contained only those time periods where the monkey was performing a single clear ambulatory behavior (e.g., epochs consisted of only bipedal walking, or only forward quadrupedal walking). Corridor and Ladder sessions consisted of walking back and forth along the corridor or ladder for a total of 20–30 rounds. These rounds contained only the steps performed at the middle of the corridor or ladder, to avoid the initiation and termination phase of locomotion.

### 2.4. Data Preprocessing

Neural data was obtained using the Blackrock Cerebus system, and saved onto a local computer through the Blackrock Central Suite program. The data was transmitted through a custom wireless headstage system (Yin et al., 2014), allowing for freely-moving recordings. The neural signal was band-passed at 500–5,000 Hz (second order Butterworth filter) and spikes were extracted by manual sorting using custom Matlab scripts (Laurens et al., 2013), which performed PCA on spike amplitudes and peak velocities (although in practice, any commonly accepted spike sorter can be used to obtain the firing rates). Neurons (the total number ranging between 18 and 80 for each session) were extracted from the multi-unit activity for each recording day (Table 1) and the spike times (counts) were binned into 10 ms intervals to match the 100 Hz kinematic data.

**Table 1:** Number of trials and neurons recorded for each recording day of each animal.

	**Neurons**	**TRM trials**	**BIP trials**	**BACK trials**	**LAD trials**	**CORR trials**	**Trials with kinematics**
Q1 Day1	56	96 (52)	43 (0)	4 (0)	35 (0)	18 (7)	59
Q1 Day2	80	91 (78)	61 (31)	4 (0)	14 (14)	5 (5)	128
Q2 Day1	63	129 (43)	12 (0)	0 (0)	6 (3)	6 (5)	51
Q2 Day2	51	217 (193)	22 (22)	0 (0)	22 (13)	4 (2)	230
Q3 Day1	46	77 (77)	6 (4)	0 (0)	10 (1)	5 (4)	86
Q3 Day2	41	79 (0)	0 (0)	0 (0)	16 (0)	12 (0)	0
Q4 Day1	51	224 (139)	0 (0)	0 (0)	0 (0)	0 (0)	139
Q4 Day2	18	51 (0)	0 (0)	0 (0)	3 (0)	20 (0)	0
Q5 Day1	39	37 (37)	0 (0)	0 (0)	26 (8)	9 (7)	52

*We were unable to obtain kinematics in some of the trials; the number of trials that did contain kinematics for each task are displayed in parenthesis, and the total number of trials that contains kinematics across all tasks is displayed in the last column. All the trials shown here contain complete neural data and gait cycle data*.

Each epoch was divided into trials consisting of a single gait cycle by manually marking the time point of foot-strike and toe-off. The stance phase of a single gait cycle was defined as the time period between the foot-strike and toe-off and represents 0–60% of the gait cycle while the swing phase was defined as the period between toe-off and the next foot-strike, and represents 60–100% of the gait cycle. The time-varying gait phase percentage was linearly interpolated from the foot-strike and toe-off time points (defined as the 0/100% and 60% mark, respectively).

Occasionally, neural data would become lost or corrupt for periods within a trial. Trials were manually inspected for data corruption, and if a trial was missing neural data, it was excluded from the analysis. All the trials shown in Table 1 had complete neural data. Additionally, for two of the recording sessions, we did not record kinematics, and for the other seven sessions where kinematics were recorded, there were occasional trials where we were unable to obtain the kinematics (for example, due to video occlusion). Table 1 contains the number of trials that contain usable kinematics for each task during each recording day. However, despite having incomplete kinematics for some of the trials, we were able to obtain gait phase data for all of the trials shown in Table 1, including in the two sessions where we were unable to obtain any kinematics. Therefore, our gait phase decoding analysis has a sample size of 9 (Figure 3C) while the kinematic decoding analysis has a sample size of 7 (Figure 3D).

### 2.5. Dimensionality Reduction Models

We used explicit state-space models to estimate the latent states of full population neural activity during locomotion. A LDS model assumes the neuronal ensemble activity as Gaussian linear observations, and uses expectation maximization (EM) learning to estimate the unknown model parameters and the latent states given only the observations (Macke et al., 2011; Aghagolzadeh and Truccolo, 2014). To account for the count process nature of ensemble spiking activity, we used a PLDS model, adding the assumption that the neural observations are conditionally Poisson given latent states (Truccolo et al., 2005; Aghagolzadeh and Truccolo, 2016). A Laplace approximation was used to compute the posterior density of the latent states given the neural observations. For decoding latent states from novel neural data, we used the mean of the state posterior density under the Laplace approximation as the estimate for the latent state (Figure 1B). The algorithm details have been described in Macke et al. (2011) and Aghagolzadeh and Truccolo (2016).

To compare PLDS with other dimensionality reduction techniques, we also tested decoders using low-dimensional inputs derived via principal component analysis (PCA), and also the activity of an optimal subsample of neurons from the full population, referred to as predictive subsampling (PSS). For PCA, the trials were concatenated across time and z-scored. The covariance matrix was computed, and the PCA projection matrix was constructed by eigenvalue decomposition—stacking the eigenvectors corresponding to the *n* largest eigenvalues, where *n* is chosen as the number of dimensions. We note that although PCA (and related methods) provide a low-dimensional representation of the ensemble activity, unlike PLDS, they do not explicitly account for any temporal dynamics in the latent states. PSS selects a subset of *n* neurons from the full population that optimize neural decoding through a greedy supervised learning algorithm, hence the name predictive. Details of this method can be found in a previous paper (Aghagolzadeh and Truccolo, 2016).

### 2.6. Comparison of Low-Dimensional Trajectories

**Figure 2:**
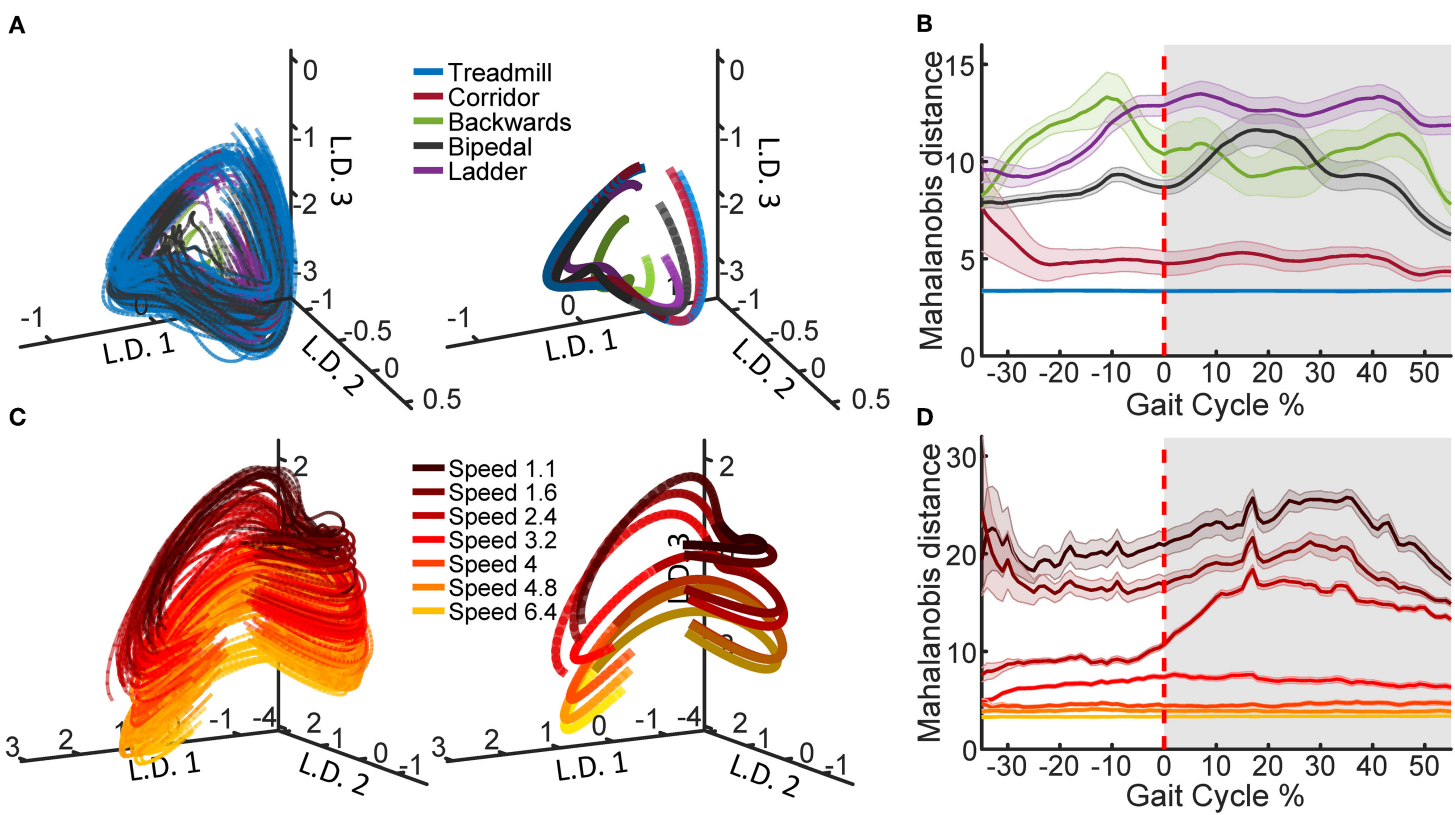
Latent state space of neural population activity during locomotion. **(A)** Neural trajectories obtained from the PLDS model for the gait cycles of all tasks in animal Q1 on recording day 1. The trajectories in the first three latent dimensions (L.D.) for each individual gait cycle are shown (left), as well as task-averaged trajectories over the total gait cycle (right). **(B)** The Mahalanobis distance between the trajectories of each of the tasks and the trajectories of the treadmill task, calculated on all 12 dimensions. Shaded region denotes stance phase (0–60% of the gait cycle), shaded bars denote 1 S.E.M. **(C)** PLDS state-space trajectories in 3 dimensions for all treadmill walking trials at different locomotion speeds in animal Q2, session 2. Darker colors represent slower speeds; all speed values displayed in the legend are in km/h. As in **(A)**, individual gait cycles (left) and gait cycles averaged for each speed (right) are shown. For the average trajectories in both **(A,C)**, the darker shade represents the stance phase while the lighter shade represents the swing phase. **(D)** The Mahalanobis distance between the trajectories of each of the speeds and the trajectory of the fastest speed (6.4 km/h).

To generate the example neural trajectories shown in Figure 2, we selected one recording session, Q1-day 1, which contained examples of all the tasks and treadmill speeds. In order to compute the average trajectories, the PLDS latent states were time-normalized to 0–100% of the gait cycle in steps of 1% using linear interpolation, with 0% representing the start of the trial (start of the stance phase) and 100% representing the last time point of the trial (end of the swing phase), while 60% was set to the transition between stance and swing. This resulted in 100 time points for each trial, and the values were averaged at each time point. The distance metric was calculated for each trial as the Mahalanobis distance between the values of that trial and the distribution of a selected reference trial type in the full 12 dimensional space. For the comparison across tasks, reference task was treadmill walking and for the comparison across speeds, the reference was the 6.4 km/h trials. The Mahalanobis distance values were averaged across trials for each of the tasks/speeds. The standard error of the mean (SEM) was also computed and shown for each task/speed.

### 2.7. Frequency Analysis

To compare the smoothness of the decoder outputs, the power spectral density (PSD) of the decoded kinematics was computed for each of the four decoders, along with the real kinematics. For each trial, we estimated the PSD using Thomson's multitaper method (*pmtm()* function in Matlab), and the values were averaged across all the trials of all the sessions. This was done for each of the decoded kinematic variables.

### 2.8. Cross-Validation

We constructed four different decoders (which we will refer to as the Full Population, PSS, PCA and PLDS decoders) and determined the performance of each through a 10-fold cross-validation paradigm (Figure 1B). The PSS, PCA and PLDS decoders employed a two-stage method where binned spike counts were projected onto a low-dimensional space, with dimensionality varying from 1 to 20. The coordinates of the neural data in this space were then utilized as input features for the Wiener filter described below. The Full Population decoder did not employ the dimensionality reduction stage and utilized the z-score normalized spike counts of all recorded neurons as input features (Figure 1A). The PLDS inputs assumes spike counts, and utilized the 10 ms bins, while the PCA, PSS, and full population utilized firing rates. The number of spikes is too sparse to estimate the firing rate using only 10 ms counts, so to estimate the firing rates at each 10 ms time step, the number of spikes in the current time bin as well as the previous nine time bins were summed and divided by 100 ms to get the rate in spikes/s. For all decoders, the time steps of the inputs was 10 ms.

Cross validation was performed on each recording session of each monkey. All of the trials of all five tasks were combined and randomized. The trials of all the tasks were then divided into 10 cross-validation blocks. Each block consisted of 5–24 (for decoding kinematics) and 7–27 (for decoding gait) trials which were used as the testing set while the remaining nine blocks were used as the training set. Decoder coefficients were calculated from the training sets using the least-squares regression algorithm between the neural data (Full Population and PSS) or latent states (PCA and PLDS) and the measured output signal (kinematics, or gait phase). The decoder was then used to estimate the output signals of the testing set, and the decoding accuracy was calculated as the coefficient of determination (R^2^) between the estimated and the true signal as defined by equation 1. *n* is the number of points of the kinematic or gait variable in the trial, y_i_ is the actual variable value at point *i*, ŷ_i_ is the estimated value from the decoder at point *i*, and ȳ is the average value of the kinematic or gait variable for the trial.

(1) $$\begin{array}{l} R^{2}=1-\frac{\sum_{i=1}^{n}{\left(y_{i}-\hat{y_{i}}\right)}^{2}}{\sum_{i=1}^{n}{\left(y_{i}-ȳ\right)}^{2}} \end{array}$$

### 2.9. Decoder

A linear Wiener filter of order 10 (the filter order that gave the best decoding results under cross validation) was used to decode the kinematics and gait cycle phase from the neural data. The decoded signals included the horizontal and vertical positions of the hip, knee, ankle, metatarsal, and toe tip joint markers, the joint angles, the leg extension, and the percentage of the gait cycle phase. The decoder is described by the equation:

(2) $$\begin{array}{l} y\left[t\right]=\overset{9}{\underset{n=0}{\sum }}A_{n}*X\left[t-n\right] \end{array}$$

where *y*[*t*] is the output signal being decoded at time *t*, and *X*[*t* − *n*] is a vector of the decoder inputs at lagged times *t* − *n*, for *n* = 0, …, 9. *A*_*n*_ is the corresponding vector of regression coefficients computed from the training set.

Statistical tests comparing the R^2^ values were carried out in Matlab (*signrank()* function).

## 3. Results

### 3.1. Structure of Population Dynamics Varies Across Different Locomotion Tasks and Speeds

Using the PLDS model, we extracted cyclic, low-dimensional neural trajectories during locomotion. Visualizing in three dimensions, the trajectories follow similar, saddle-like rotations across different tasks, and across different walking speeds (Figure 2A). The Mahalanobis distance between all of the task trajectories against just the treadmill walking trajectories demonstrates that the corridor walking trajectories are the most similar to treadmill walking (mean distance = 5.0392), followed by bipedal, backwards, and ladder walking in various orders depending on the phase of the gait (mean distance = 9.12, 10.66, and 11.97, respectively, Figure 2B). However, any interpretation of the neural trajectories for backwards walking should be treated with caution due to the low number of trials available (Table 1). When training the PLDS model only on treadmill walking trials, there is a similar rotational structure across all walking speeds, however trajectories appear to separate along the third latent dimension (Figure 2C). The change in state-space position along this dimension is reflected in the increase in Mahalanobis distance between trials of different speeds as the difference in speed increases (Figure 2D). In general, the PLDS model was able to infer latent state trajectories that are closely phase-locked to the locomotor rhythm, and distinguishes relevant behavioral parameters such as task type and walking speed. We next describe how well kinematic and gait parameters could be decoded from these low-dimensional dynamics.

### 3.2. PLDS Latent State Trajectories Capture Limb Kinematics and Gait Phase

**Figure 3:**
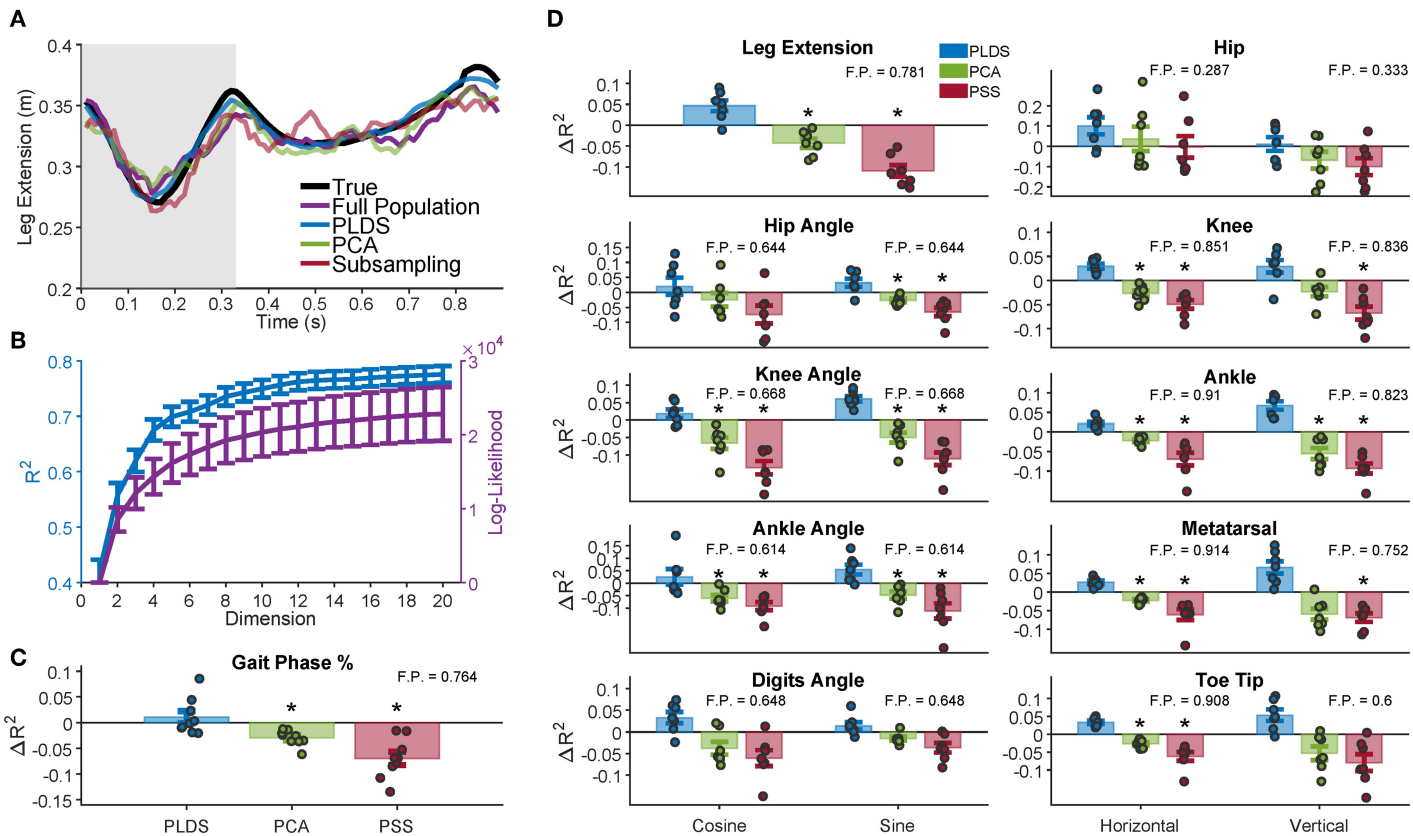
Decoding of leg kinematics and gait phase. **(A)** A representative example of the limb extension kinematic variable during one gait cycle along with the decoded estimation for the four decoders. Gray background represents the swing period of the gait cycle, white background represents the stance period. In this example, the PLDS decoded signal was the most successful at reconstructing the true signal, with an R^2^ of 0.8294, followed by decoding without any dimensionality reduction (Full Population: FP) with an R^2^ of 0.5461. PCA and PSS decoding performed the worst, with R^2^ of 0.5355 and 0.4980, respectively. The R^2^ values for all of the animals and sessions are shown in **(D)**. **(B)** Dimensionality analysis for PLDS reveals a plateau in decoding performance (blue) at approximately 12 dimensions for the latent states (plateau defined as when the increase in R^2^ <0.001), error bars 1 SEM. Log-likelihood (purple) also plateaus at around 12 dimensions. **(C)** Cross-validated decoding performance of dimensionality reduction techniques for decoding gait phase percentage. Bars represent the increase in R^2^ over the Full Population decoder, with the inset number representing the average R^2^ of the Full Population decoder. The full distribution of change in R^2^ are displayed as dots over each bar. The PLDS decoder had the highest average R^2^ for all the variables. Error bars: 1 S.E.M. Black stars denote significant difference from Full Population decoding R^2^ (Wilcoxon Sign Rank Test; Benjamini-Hochberg false discovery rate correction for multiple comparisons, with FDR = 10%). **(D)** Same as in **(C)**, except for all analyzed kinematic variables rather than gait phase. Each dot represents a animal/recording session, and the bar plots show the average R^2^ with error bars representing 1 S.E.M.

We used several neural features (full population firing rates, PLDS or PCA latent variables, or a predictive sub-sample (PSS) of the population firing rates) as inputs to a Wiener filter decoder and measured how well each input feature could decode various kinematic variables under cross-validation. To determine the total number of dimensions to use in our decoders, we measured the PLDS decoder performance for all the kinematic variables while varying the input dimensionality from 1 to 20. We found that the performance plateaued at approximately 12 dimensions (Figure 3B); we used this number of dimensions for the remainder of the analyses, as well as for calculating the Mahalanobis distances in Figure 2.

First, we investigated whether using PLDS as a feature extractor would improve or deteriorate decoding of hind-limb kinematics. An example trace of the decoded leg extension for one trial is shown in Figure 3A. For this example, the estimate from the PLDS decoder approximates the true signal more faithfully than the estimate from the Full Population decoder (*R*^2^ = 0.8294 vs. *R*^2^ = 0.5461), as well as compared to the PCA and PSS decoders (*R*^2^ = 0.5355 and *R*^2^ = 0.4980, respectively). We then compared the cross-validation results for the various decoders across all monkeys and recording sessions that have kinematics (*n* = 7) for each of the kinematic variables, including the horizontal and vertical position of the joints, the joint angles (taking the sine and cosine to ensure valid *R*^2^ values), and leg extension (two-tailed Wilcoxon signed-rank test, Benjamini-Hochberg false discovery rate correction for multiple comparisons, with FDR = 10%). The PLDS decoder performance was not significantly different from the full-population decoder for any of the tested kinematic variables despite having only 12 input features, and indeed for all variables, the average PLDS decoder R^2^ was higher than the full population, although none of them reached significance when corrected for multiple testing (Figure 3D). However, the PCA decoder performed worse than the full population for many of the kinematic variables, as did the PSS decoder (see Table S1 for *p*-values), leading us to conclude that PCA and PSS features, at the same number of dimensions, do not preserve the kinematic information as completely as those obtained with the PLDS model. From the sample trial in Figure 3A, the PLDS decoded signal appears much smoother than the other three estimations, which may account for the higher R^2^. The power spectrum of the decoder outputs verifies that the PLDS decoder and the real kinematics have less power at high frequencies compared to the full population, PCA, and PSS decoders (Figure S1). Although the Wiener filter does provide some degree of smoothing by taking into account the previous 10 time bins in the decoding, the PLDS model smooths the intrinsic trajectories based on the activity of the whole ensemble, which may provide a more accurate estimation of the dynamics.

In addition to the above kinematic variables, we also investigated how well gait information, such as the phase of the gait cycle, could be estimated using each decoder. Trials consisted of a single gait cycle, and the phase of each cycle were standardized to a range of 0–100%. The phase was then estimated by each of the decoders, and the change in R^2^ from the full population decoder is shown in Figure 3C for each of the recording sessions (*n* = 9). The decoding performance was similar to the decoding of kinematics; there was no significant difference in R^2^ between the PLDS decoder and full population decoder, and the PCA and PSS decoder performed worse than the full population decoder (see Table S1 for *p*-values).

## 4. Discussion

While the PLDS and PCA models are both dimensionality reduction techniques, a key difference of PLDS is it explicitly models the temporal dynamics using a linear dynamical system. Other techniques have been developed which also models these dynamics such as jPCA (Churchland et al., 2012), or Hypothesis-guided dimensionality reduction (HDR) (Lara et al., 2018). These other techniques introduces some constraints to the form of the dynamical system in order to test specific hypotheses about the structure of cortical dynamics. For example, jPCA limits the temporal transition matrix to the set of skew-symmetric matrices in order to extract rotational dynamics, while in Lara et al. (2018), HDR was used to divide the projected dimensions into linear dynamical systems states and dimensions orthogonal to those states that are condition-invariant (in order to find similarities between two different brain regions). We did not wish to impose any additional constraints about the form the dynamical systems so we used the general form PLDS model for this analysis.

Both the PCA and the PLDS model extracted neural trajectories that were oscillatory in low-dimensional space. PLDS explicitly models temporal dynamics of the latent states with a state transition matrix, resulting in smoother single-trial cyclical trajectories (Figure 1B, Figure S1). The structure of the trajectories for treadmill walking at different speeds are in agreement with previous studies, where the neural latent-states separated along one dimension as walking speeds increased while still conserving the rotational structure (Foster et al., 2014). We were also able to obtain qualitatively distinct neural trajectories during different tasks (Figure 2A), although again, rotational structure is preserved across all tasks.

The dimensionality of neural data required to represent hind-limb kinematics was approximately 12 dimensions, when empirically determined as the plateau in kinematic decoding performance (Figure 3B). Previous studies have estimated the intrinsic dimensionality in forelimb motor cortex during center out tasks to be around 10–20 dimensions (Yu et al., 2009; Sadtler et al., 2014; Vargas-Irwin et al., 2015). Despite the more constrained movements of the hind-limb during locomotion, the dimensionality between leg and arm area of M1 and between reaching and walking are surprisingly similar. Such similarity could suggest comparable levels of cortical involvement during these different behaviors (although the actual structure of the cortical activity could be different). In 1989, Georgopoulos and Grillner proposed the hypothesis that reaching movements in primates may have evolved out of precise gait adjustments during locomotion (Georgopoulos and Grillner, 1989), and others have suggested that similarities in neural and muscle activation onsets between those two types of movements support this hypothesis (Yakovenko and Drew, 2015). The similarities in neural dimensionality between voluntary reaching and walking would also be consistent with this view. However, due to the low sample size and the use of only one dimensionality reduction technique in this study, further experiments exploring additional models may be necessary before any definitive conclusions can be drawn on the dimensionality of leg-M1 population activity during locomotion.

It is well understood that neurons in motor cortex are correlated to muscle activity and kinematics during locomotion in cats (Beloozerova and Sirota, 1998; Drew et al., 2002; Drew and Marigold, 2015), rodents (Song et al., 2009; Rigosa et al., 2015; DiGiovanna et al., 2016; Miri et al., 2017), and non-human primates (Fitzsimmons et al., 2009; Foster et al., 2014; Yin et al., 2014). Here, we showed whether the extracted low dimensional dynamics from PLDS preserve the kinematic information that is present in the neural activity. In terms of the decoding performance, PLDS with only 12 dimensions was able to reconstruct all the kinematic variables in addition to the gait phase just as well as the full population decoder that contains 18–80 dimensions. There is some variance in the distribution of PLDS decoder improvements across animals and sessions (Figure 3). This could be due to the different populations of neurons that are recorded from in different subjects. These populations are not homogeneous and may represent the true underlying cortical dynamics by varying degrees. Other dimensionality reduction techniques such as PCA or PSS were unable to achieve the same decoding performance at the same number of dimensions, indicating that an explicit model of temporal dynamics of the low-dimensional states, such as PLDS, is crucial for decoding the kinematics accurately. These results reflect the improved decoding performance using linear dynamical systems models during cursor control from arm area (Yu et al., 2009; Kao et al., 2015) and during reaching and grasping behaviors (Aghagolzadeh and Truccolo, 2016), suggesting that low-dimensional dynamics play an important role in both types of movements. Our findings differ in that we did not see a statistically significant increase in performance when using latent state input features, whereas the arm decoding studies did see an improvement. However, given that the R^2^ of every single kinematic variable was on average higher with the PLDS decoder compared to the full population decoder, this may be due to our low sample size and lack of statistical power. Additionally, the power is lowered by the use of parametric tests and the large number of kinematic variables tested.

We should also mention that our decoders utilized firing rate inputs, and thus carries the assumption of rate coding rather than temporal coding. Recent findings have suggested that temporal coding may play a larger role in motor control than previously thought (Srivastava et al., 2017). One future extension of this study would be to include models that utilize precise spike timings. Additionally, the Wiener filter decoder we employed is a linear decoder, and although it was able to reconstruct the kinematics fairly accurately, other non-linear models could be used to further improve decoding performance. Finally, we should mention that this study was limited to higher-level control areas such as the motor cortex, though lower-level structures such as brain-stem or spinal cord have been shown to also exhibit intrinsic population dynamics (Bruno et al., 2017).

In conclusion, our study investigates whether unsupervised dimensionality reduction can infer latent neural states reflecting ensemble dynamics, while preserving information about the kinematics and gait phase of the hind-limb during various locomotion tasks. We show that dynamical systems models, which have been shown to decode forelimb reaching kinematics, were able to extract robust, stereotyped low-dimensional state-space trajectories, and that these trajectories capture hind-limb movements during directed locomotion (e.g., ladder walking), as well as autonomous locomotion (e.g., basic treadmill and corridor walking). As far as we are aware, this is the first demonstration of explicit state-space models of neural dynamics robustly decoding kinematic and gait information during primate locomotion. These results also points to the potential of using PLDS in hind-limb BMIs, although direct testing in a closed-loop system would be required before any determination of the usefulness of PLDS as a feature extraction step can be made. Recently, newer techniques have been developed to extract neural dynamics using recurrent neural networks (Pandarinath et al., 2018) which enable extraction of non-linear dynamics and have been employed in arm reaching tasks. Such models may extract the underlying neural dynamics more accurately and may outperform PLDS in terms of decoding of kinematics. One potential extension to this study in the future would be to apply these non-linear models to hind-limb locomotion behaviors as well.

## Data Availability Statement

Data are available by directly contacting GC at gregoire.courtine@epfl.ch.

## Ethics Statement

The animal study was reviewed and approved by Institutional Animal Care and Use Committee of Bordeaux (CE50), license number 50120102-A.

## Author Contributions

DB, EB, and GC performed data collection experiments. DX and MA performed decoding analysis. WT and DB conceived of and oversaw the project. EB and GC oversaw the original data collection. DX wrote the manuscript with MA. All authors contributed to the editing and finalization of the manuscript.

### Conflict of Interest

The authors declare that the research was conducted in the absence of any commercial or financial relationships that could be construed as a potential conflict of interest.
