# Corrigendum: Low-Dimensional Motor Cortex Dynamics Preserve Kinematics Information During Unconstrained Locomotion in Nonhuman Primates

**DOI:** 10.3389/fnins.2020.604517

**Published:** 2020-10-29

**Authors:** David Xing, Mehdi Aghagolzadeh, Wilson Truccolo, Erwan Bezard, Gregoire Courtine, David Borton

**Affiliations:** ^1^School of Engineering, Brown University, Providence, RI, United States; ^2^Department of Neuroscience, Brown University, Providence, RI, United States; ^3^Carney Institute for Brain Science, Brown University, Providence, RI, United States; ^4^U.S. Department of Veterans Affairs, Center for Neurorestoration and Neurotechnology, Providence, RI, United States; ^5^CNRS, Institut des Maladies Neurodégénératives, UMR 5293, Bordeaux, France; ^6^Université de Bordeaux, Institut des Maladies Neurodégénératives, UMR 5293, Bordeaux, France; ^7^Center for Neuroprosthetics and Brain Mind Institute, School of Life Sciences, Swiss Federal Institute of Technology (EPFL), Geneva, Switzerland; ^8^Department of Clinical Neuroscience, Lausanne University Hospital (CHUV), University of Lausanne (UNIL), Lausanne, Switzerland; ^9^Defitech Center for Interventional Neurotherapies (NeuroRestore), CHUV/UNIL/EPFL, Lausanne, Switzerland; ^10^Department of Neurosurgery, CHUV, Lausanne, Switzerland

**Keywords:** low dimensional dynamics, locomotion, non-human primate (NHP), poisson linear dynamical system, primary motor cortex (M1)

In the original article, we neglected to include the funder Marie Curie Fellowship, 331602 (e-WALK) to DB under the direct mentorship of GC.

The Funding section has been updated to read as follows:

This work was sponsored by the Defense Advanced Research Projects Agency (DARPA) BTO under the auspices of Dr. Doug Weber and Alfred Emondi through the [Space and Naval Warfare Systems Center, Pacific OR DARPA Contracts Management Office] Grant/Contract No. D15AP00112; the DARPA program Neural Engineering Systems Design (NESD) (N666001-17-C-4013); as well as the International Research in Paraplegia Foundation (IRP, P152). This work was also supported by Marie Curie Fellowship, 331602 (e-WALK) to DB under the direct mentorship of GC. We would also like to acknowledge the Pablo J. Salame '88 Goldman Sachs endowed Associate Professorship of Computational Neuroscience at Brown University (WT), and the Howard Reisman '76 Family Graduate Fellowship Fund and the Charles A. Dana Graduate Fellowship Fund (DX). This work was supported in part by Merit Review Award # I01RX002835 from the United States (U.S.) Department of Veterans Affairs, Rehabilitation Research and Development Service. The contents of this manuscript do not represent the views of VA or the United States Government. Department of Veterans Affairs did not provide any direct support of the NHP work conducted for this project. These funding sources had no involvement in the design of this study, collection or analysis of data, nor in the authorship of this manuscript.

Additionally, in the original article, the Data Availability Statement referenced the incorrect person/institution to contact for data access. A correction has been made to the Data Availability section to now read:

Data are available by directly contacting GC at gregoire.courtine@epfl.ch.

Finally, in the original article, the authorship did not reflect the contributions of members who supported the study. The authorship and the authorship contributions have been changed to now read:

David Xing^†1^, Mehdi Aghagolzadeh^2†^, Wilson Truccolo^2,3,4^, Erwan Bezard^5,6^, Gregoire Courtine^7,8,9,10‡^ and David Borton^1,3,4*‡^

DB, EB, and GC performed data collection experiments. DX and MA performed decoding analysis. WT and DB conceived of and oversaw the project. EB and GC oversaw the original data collection. DX wrote the manuscript with MA. All authors contributed to the editing and finalization of the manuscript.

The authors apologize for these errors and state that they do not change the scientific conclusions of the article in any way. The original article has been updated.

