# Supplementary material for: Low-Dimensional Motor Cortex Dynamics Preserve Kinematics Information During Unconstrained Locomotion in Nonhuman Primates
Source: Front Neurosci. 2019 Oct 4;13:1046. doi: 10.3389/fnins.2019.01046 (PMC6788380; doi:10.3389/fnins.2019.01046)
Supplement: Supplementary file 1 [file Data_Sheet_1.PDF]

Supplementary Material

	PLDS	PCA	PSS
Gait phase	0.7344	0.0039	0.0039
Leg extension	0.0313	0.0156	0.0156
Hip angle cosine	0.5781	0.3750	0.0781
Hip angle sine	0.1094	0.0156	0.0156
Knee angle cosine	0.2188	0.0156	0.0156
Knee angle sine	0.0156	0.0156	0.0156
Ankle angle cosine	0.5781	0.0156	0.0156
Ankle angle sine	0.0313	0.0156	0.0156
Digits angle cosine	0.0469	0.0781	0.0313
Digits angle sine	0.1094	0.0313	0.0313
Hip horizontal position	0.0781	0.5781	1.0000
Hip vertical position	0.5781	0.2969	0.0781
Knee horizontal position	0.0156	0.0156	0.0156
Knee vertical position	0.1563	0.0469	0.0156
Ankle horizontal position	0.0156	0.0156	0.0156
Ankle vertical position	0.0156	0.0156	0.0156
Metatarsal horizontal position	0.0156	0.0156	0.0156
Metatarsal vertical position	0.0156	0.0313	0.0156
Toe tip horizontal position	0.0156	0.0156	0.0156
Toe tip vertical position	0.0313	0.0469	0.0313

Table S1. p-values for each of the kinematic and gait variable tested for each decoder (compared to full-population decoder). Wilcoxon signed rank test with null hypothesis that there is no difference in the distribution between the full-population decoder R^2s and the tested decoder R^2s. Significance was tested for after applying Benjamini-Hochberg false discovery rate correction for multiple comparisons, with FDR=10%

.

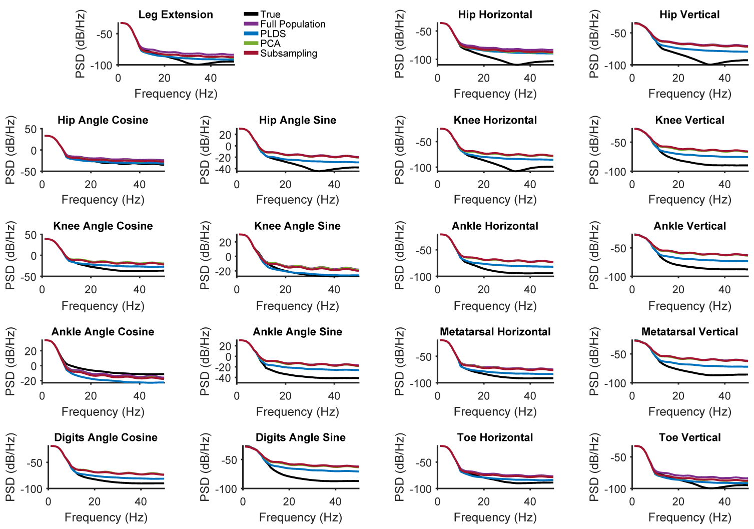

Figure S1. Power spectral density of the decoded kinematics for each of the decoders, as well as the real kinematics (black trace). The PSD is shown for each of the kinematic variables in Figure 3d
